# The UBAP1 Subunit of ESCRT-I Interacts with Ubiquitin via a SOUBA Domain

**DOI:** 10.1016/j.str.2011.12.013

**Published:** 2012-03-07

**Authors:** Monica Agromayor, Nicolas Soler, Anna Caballe, Tonya Kueck, Stefan M. Freund, Mark D. Allen, Mark Bycroft, Olga Perisic, Yu Ye, Bethan McDonald, Hartmut Scheel, Kay Hofmann, Stuart J.D. Neil, Juan Martin-Serrano, Roger L. Williams

**Affiliations:** 1 Department of Infectious Diseases, King's College London School of Medicine, London SE1 9RT, UK; 2 MRC Laboratory of Molecular Biology, Cambridge CB2 0QH, UK; 3 Miltenyi Biotec, 51429 Bergisch-Gladbach, Germany

## Abstract

The endosomal sorting complexes required for transport (ESCRTs) facilitate endosomal sorting of ubiquitinated cargo, MVB biogenesis, late stages of cytokinesis, and retroviral budding. Here we show that ubiquitin associated protein 1 (UBAP1), a subunit of human ESCRT-I, coassembles in a stable 1:1:1:1 complex with Vps23/TSG101, VPS28, and VPS37. The X-ray crystal structure of the C-terminal region of UBAP1 reveals a domain that we describe as a solenoid of overlapping UBAs (SOUBA). NMR analysis shows that each of the three rigidly arranged overlapping UBAs making up the SOUBA interact with ubiquitin. We demonstrate that UBAP1-containing ESCRT-I is essential for degradation of antiviral cell-surface proteins, such as tetherin (BST-2/CD317), by viral countermeasures, namely, the HIV-1 accessory protein Vpu and the Kaposi sarcoma-associated herpesvirus (KSHV) ubiquitin ligase K5.

## Graphical abstract

**Graphical Abstract:**

**Figure:**
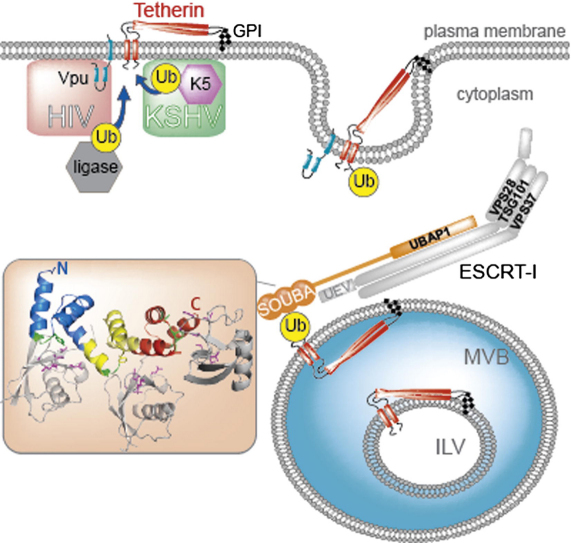

**Highlights:**

► ESCRT-I subunit UBAP1 is essential for degradation of antiviral protein tetherin ► UBAP1 has a domain consisting of a solenoid of overlapping UBAs (SOUBA) ► Each of the three UBAs in the SOUBA binds monoubiquitin

## Introduction

The endosomal sorting complex required for transport (ESCRT) machinery facilitates the lysosomal degradation of ubiquitinated cell surface receptors (Hurley and Stenmark, 2011; Katzmann et al., 2001). ESCRT proteins are conserved from yeast to humans and form four multiprotein complexes termed ESCRT-0, ESCRT-I, ESCRT-II, and ESCRT-III (Henne et al., 2011; Williams and Urbé, 2007). ESCRT-0, -I, and -II capture ubiquitinated membrane proteins for sorting into intraluminal vesicles (ILV) within endosomes to form structures known as multivesicular endosomes or multivesicular bodies (MVB) (Shields and Piper, 2011).

The ESCRT machinery is also essential for resolution of the midbody during cytokinetic abscission (Carlton and Martin-Serrano, 2007; Morita et al., 2007b), a process that is topologically equivalent to MVB formation. The ability of the ESCRTs to mediate scission of a thin membranous stalk is also exploited by several enveloped viruses such as HIV-1 to facilitate their release from infected cells (Baumgärtel et al., 2011; Jouvenet et al., 2011; Martin-Serrano and Neil, 2011; Morita and Sundquist, 2004; Morita et al., 2011; Weissenhorn and Göttlinger, 2011). In particular, HIV-1 encodes a PTAP motif that recruits ESCRT-I to the sites of viral budding through a direct interaction with the UEV domain in TSG101 (Pornillos et al., 2002). Additional roles of ESCRT-I in viral pathogenesis include its cooption by gamma-herpesviruses (Nathan and Lehner, 2009) and HIV for the degradation of various antiviral cell-surface proteins such as tetherin (BST-2/CD317) (reviewed in Martin-Serrano and Neil, 2011). Tetherin is an antiviral type II membrane glycoprotein that is induced by interferons and physically inhibits enveloped virus particle release from infected cells by cross-linking nascent virions to the plasma membrane. Specifically, the HIV-1 accessory protein Vpu counteracts tetherin activity and promotes its ESCRT-dependent degradation via the lysosomal pathway (Janvier et al., 2011). Kaposi sarcoma-associated herpesvirus (KSHV) encodes K5, a membrane-bound E3 ubiquitin ligase that results in a similar effect (Bartee et al., 2006; Mansouri et al., 2009; Pardieu et al., 2010).

ESCRT-I is formed by four subunits, Vps23/TSG101, Vps28, Vps37, and Mvb12. The yeast ESCRT-I heterotetramer contains a fan-shaped headpiece formed by a heterotrimeric core consisting of the C-terminal “steadiness box” of Vps23p, the N-terminal half of Vps28p, and the C-terminal half of Vps37p (Kostelansky et al., 2006; Teo et al., 2006). This headpiece connects to an extended stalk formed by Mvb12p, Vps23p, and Vps37p. The stalk is essential for yeast ESCRT-I function in cargo sorting. The C-terminal domain of Vps28p is flexibly tethered to the headpiece and binds ESCRT-II, whereas the flexibly attached UEV domain of Vps23p binds to ESCRT-0 (Kostelansky et al., 2007). This structural organization is thought to be conserved in mammalian ESCRT-I and was used as the basis to identify, to our knowledge, novel subunits of the complex. However, in mammalian cells, ESCRT-I has evolved a much greater diversity of subunits than in yeast, including multiple isoforms of VPS37 and MVB12 (Bache et al., 2004; Eastman et al., 2005; Morita et al., 2007a; Stuchell et al., 2004).

**Figure 1:**
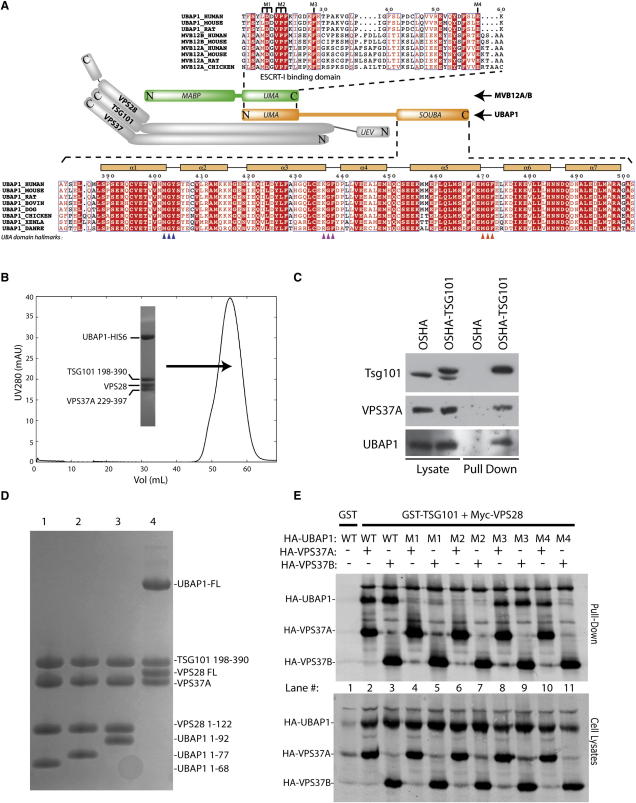
UBAP1 Forms a Stable Heterotetrameric Complex with ESCRT-I Subunits TSG101, VPS28, and VPS37 In Vitro and In Vivo (A) Schematic representation of the ESCRT-I heterotetramer illustrating the putative UBAP1 arrangement relative to other ESCRT-I subunits. The top sequence alignment shows the UMA domain, also present in the C-terminal part of MVB12A and MVB12B. The bottom part shows a detailed view of the conserved sequence corresponding to the SOUBA domain in the C-terminal region of UBAP1 from various species. Hallmark UBA residues homologous to the conserved (M/L)-G-(Y/F) motif for the three successive UBA domains are identified by blue, pink, and orange triangles, respectively. Positions of the seven α helices mapped from the structure are shown above the alignment. (B) Gel filtration of recombinant ESCRT-I containing UBAP1-His6 on a HiLoad 16/60 Superdex 200 column. The inset shows a Coomassie blue-stained SDS-PAGE gel of the peak fraction containing the tetrameric ESCRT-I complex including UBAP1. See also Table S1 and Figure S1. (C) ESCRT-I protein complexes from cells stably expressing One-Strep tagged TSG101 (OSHA-TSG101) were affinity purified on a Strep-Tactin matrix and visualized by western blot. One percent of the starting cell lysate and 10% of the volume eluted from the matrix (pull-down) were analyzed by western blot with anti-TSG101, anti-VPS37A, and anti-UBAP1 antibodies. As a control for the specificity of the binding, purification from cells stably expressing an empty vector (OSHA) is also shown. (D) Deletion analysis to determine the minimal ESCRT-I binding region on UBAP1. Recombinant UBAP1, either full length (lane 4) or N-terminal fragments (1–92, lane 3; 1–77, lane 2; or 1–68, lane 1), with a His6 tag at the C terminus were coexpressed with TSG101/VPS28/VPS37A ESCRT-I components in *E. coli* and purified by affinity chromatography and gel filtration. A Coomassie-stained SDS-PAGE of the purified complexes is shown. (E) Coprecipitation studies to map the interaction of UBAP1 with ESCRT-I. 293T Cells were transfected with plasmids expressing GST-TSG101, Myc-VPS28, HA-VPS37A/B, and HA-UBAP1 wild-type (WT) or mutant (M1 to M4), followed by purification using glutathione-coated beads. One percent of the starting cell lysate and 10% of the volume eluted from the beads (pull-down) were analyzed by western blot with anti-HA antibody. See also Figure S1 and Table S1.

Using our sensitive generalized profile method for sequence comparison (Bucher et al., 1996), we identified a highly significant relationship between a profile constructed from different vertebrate and invertebrate MVB12 sequences and the protein UBAP1. An independent report also predicted a shared domain between UBAP1 and MVB12 that was named UBAP1-MVB12-associated (UMA) domain (de Souza and Aravind, 2010) (Figure 1A). The UMA domain corresponds to a region that was previously described as the ESCRT-I binding box (EBB) (Morita et al., 2007a) and is located in the C terminus of MVB12 and in the N terminus of UBAP1. UBAP1 is expressed in a wide range of tissues and is embryonic lethal when deleted in mice (http://www.sanger.ac.uk/mouseportal/search?query=ubap1). It is part of a locus that experiences a loss of heterozygosity in human nasopharyngeal cancers (Qian et al., 2001). Recently, UBAP1 was identified as a risk factor in familial frontotemporal lobar dementia (FTLD) (Rollinson et al., 2009).

In contrast to the MVB12A and MVB12B subunits, which have an N-terminal extension consisting of a MABP β-prism domain (de Souza and Aravind, 2010), UBAP1 has a greatly expanded C-terminal region, which contains three putative ubiquitin associated (UBA) domains. A number of ubiquitin-binding domains (UBDs) have been described, including CUE, UIM, NZF, UEV, GLUE, GGA, GAT, and UBA (Dikic et al., 2009). The UBA domain is the most frequently occurring UBD, with 84 sequences in the human genome according to Pfam (http://pfam.sanger.ac.uk). UBAs have about 45 residues folded into a compact three-helix bundle with a right-handed twist. UBA domains have a conserved signature motif M/L-G-Y/F in the α1/α2 loop that forms part of the interaction with ubiquitin, and there is a conserved di-leucine motif at the end of helix α3 (Hofmann and Bucher, 1996; Mueller and Feigon, 2002; Ohno et al., 2005). UBAs have been grouped in four classes according to their preference for polyubiquitin linkages: K48-linked polyubiquitin, K63-linked polyubiquitin, promiscuous recognition of multiple polyubiquitin linkages, and no ubiquitin recognition (Raasi et al., 2005). UBA domains typically also bind monoubiquitin, but generally with much lower affinity.

UBAs have important roles in many different contexts, including diverse degradative pathways. Several UBA-containing proteins, such as Rad23 and Dsk2, function as adaptors for proteasome degradation (Clague and Urbé, 2010). Degradation of polyubiquitinated protein aggregates depends on their recruitment to autophagosomes by the UBA domain of p62 (Pohl, 2009). The ESCRT complexes involved in MVB sorting in yeast contain a number of UBDs, including UIMs, VHS, NZF, and UEV domains (Shields and Piper, 2011). However, disruption of the individual UBDs in the ESCRT complexes does not block the MVB pathway and only a quadruple mutant that is completely defective for ubiquitin binding shows severe sorting defects (Shields et al., 2009). Intriguingly, UBA domains are absent from the MVB pathway in yeast.

We report here the structure of the C-terminal region of UBAP1 that folds as a solenoid of overlapping UBAs (SOUBA) domain. NMR data show that each of the three UBA domains composing SOUBA binds monoubiquitin. This is consistent with the specific role of UBAP1 in endosomal sorting of ubiquitinated membrane cargo as shown recently for EGFR by Woodman and colleagues (Stefani et al., 2011). We show that UBAP1 is required for ubiquitin-dependent degradation of the antiviral protein tetherin triggered by the viral countermeasures HIV-I protein Vpu and KSHV viral protein K5.

## Results

### Composition and Stoichiometry of ESCRT-I Complexes Containing UBAP1

Recombinant full-length human UBAP1, when coexpressed in *Escherichia coli* together with ESCRT-I subunits VPS28, TSG101, and VPS37A, forms a stable complex with 1:1:1:1 stoichiometry of the four subunits (Figure 1B; Figure S1 available online). The cores of VPS37A, VPS37B, VPS37C, or VPS37D all give rise to stable recombinant ESCRT-I complexes with UBAP1 in *E. coli* (Figure S1A). Endogenous UBAP1 from HEK293 cell lysate eluted close to the 440 kDa marker in a complex containing TSG101 (Figure S1B). In addition, endogenous UBAP1 was efficiently copurified with TSG101 and VPS37A from a cell line stably expressing TSG101 with a One-Strep tag (OSHA-TSG101) (Figure 1C). The OSHA-TSG101 fusion is expressed at endogenous levels in these cells (McDonald and Martin-Serrano, 2008), allowing affinity purification of TSG101-binding proteins under physiological conditions. Similarly, endogenous TSG101 also bound UBAP1 and VPS28 when these proteins were overexpressed in 293T cells as GST fusions and purified with glutathione beads (Figure S1C). As observed for other ESCRT-I subunits, UBAP1 functionally recruits the ESCRT machinery in a trans-complementation assay based on the rescue of a L-domain defective HIV-1 by direct fusion of a component of the ESCRT machinery to a defective Gag protein (Martin-Serrano et al., 2001). Expression of UBAP1 fused to the C terminus of the complementing Gag protein restored infectious virus production (Figures S1E–S1H).

Deletion analysis using recombinant proteins showed that the N-terminal fragment of UBAP1 comprising residues 1 to 68 is sufficient to form a stable complex with TSG101, VPS28, and VPS37A (Figure 1D). This N-terminal construct corresponds to the UMA domain (Figure 1A). Overexpression of UMA domain constructs YFP-UBAP1_1-68_ and YFP-UBAP1_1-92_ in 293T cells transfected with HIV-1 proviral DNA has a dominant negative effect, resulting in approximately 5-fold inhibition of HIV-1 infectious virus release (Figure S1D). This suggests that overexpression of the UBAP1's ESCRT-I binding region disrupts the stoichiometric composition or proper localization of endogenous ESCRT-I, thus inhibiting HIV-1 budding, as previously shown for TSG101 (Martin-Serrano et al., 2003).

To identify UMA residues necessary for interaction with other ESCRT-I subunits, we tested point mutants in several conserved residues of this domain for their ability to form ESCRT-I complexes (UMA mutants M1 to M4 in Figure 1A). Given that yeast Mvb12 is involved in direct contact with Vps37 (Kostelansky et al., 2007), we tested the ability of UMA mutants to assemble into ESCRT-I complexes containing either VPS37A or VPS37B. Cells were transfected with plasmids expressing GST-TSG101, Myc-VPS28, HA-VPS37A/B, and HA-UBAP1, followed by purification using glutathione-coated beads and immunodetection of tagged ESCRT-I subunits. Alanine substitutions L_17_D_18_D_19_/AAA and V_20_P_21_F_22_/AAA (mutants M1 and M2) led to a complete block in ESCRT-I binding (Figure 1E, lanes 4 to 7), whereas F28A (mutant M3) did not (Figure 1E, lanes 8 and 9). Interestingly, mutation of the conserved Glu-59 (E59A, mutant M4) resulted in loss of binding to ESCRT-I when HA-VPS37B was cotransfected but not when HA-VPS37A was present in the protein complex (Figure 1E, lanes 10 and 11), suggesting that the VPS37 subunits interact directly with UBAP1. These results were confirmed with the HIV-1 trans-complementation assay, showing that fusing UBAP1 constructs bearing the mutations M1, M2, or M4 to Gag did not restore release of a budding deficient HIV-1 as compared to wild-type UBAP1 or M3, indicating that these residues are needed for UBAP1's interaction with ESCRT-I components (Figure S1H).

### UBAP1 Functions in MVB Sorting of the Antiviral Protein Tetherin

**Figure 2:**
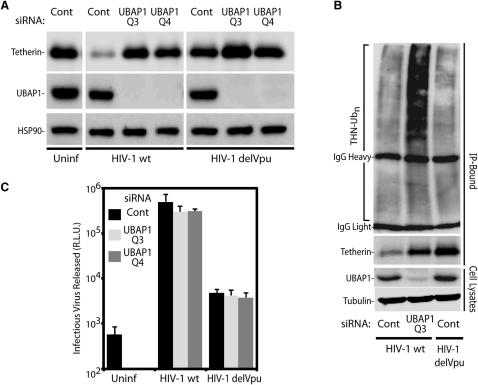
UBAP1 Is Essential for Degradation of Tetherin Triggered by Viral Countermeasures (A) Tetherin was immunoprecipitated from 293T cells stably expressing tetherin. The cells were uninfected (uninf), infected with HIV-1 (HIV-1 wt), or Vpu-defective HIV-1 (HIV-1 delVpu) and were treated with irrelevant control siRNA (Cont) or with siRNAs against UBAP1 (UBAP1Q3 and UBAP1Q4). Western blots show the immunoprecipitated tetherin (upper panel), siRNA-mediated silencing of the endogenous UBAP1 (middle panel), and the HSP90 as a protein loading control (lower panel). (B) Tetherin was immunoprecipitated from cells infected with HIV-1 (HIV-1 wt) and transfected with a plasmid expressing HA-tagged Ubiquitin and either control (cont) or UBAP1 specific siRNA (UBAP1 Q3). Tetherin ubiquitination was visualized with anti-HA antibody (top panel, THN-Ubn). As a control, the same analysis was performed in cells infected with a Vpu-defective HIV-1 (HIV-1 delVpu) and treated with an irrelevant siRNA (Cont). Bottom panels show immunoprecipitated tetherin, UBAP1 depletion and tubulin as a loading control. (C) Infectious HIV-1 virion production was measured by inoculation of TZM-bl indicator cells and is expressed as relative luminescence units (R.L.U.). Error bars indicate the standard deviation from the mean of three independent experiments. See also Table S2.

Given the role of the ESCRT machinery in sorting ubiquitinated membrane proteins, we tested the function of UBAP1 in this process. We took advantage of the ability of several viruses to reduce cell surface expression of the antiviral protein tetherin in infected cells via the ESCRT machinery. The first experimental approach is based on HIV-1's Vpu capacity to mediate tetherin degradation. Thus, 293T cells stably expressing human tetherin (293T/tetherin) were treated with UBAP1-specific siRNA or a nontargeting control and subsequently were infected with either HIV-1 (HIV-1 wt) or Vpu-defective HIV-1 (HIV-1 delVpu) as a control. Tetherin was then immunoprecipitated, deglycosylated, and visualized by western blot. As expected, cells infected with HIV-1 wild-type have greatly reduced levels of tetherin (Figure 2A). However, this tetherin degradation was prevented in UBAP1-depleted cells infected with HIV-1 wild-type virus, and a large amount of tetherin was detected in these cells. This tetherin in UBAP1-depleted, HIV-1-infected cells is highly ubiquitinated (compare the first and second lanes in Figure 2B), suggesting that the ubiquitin-dependent sorting by the MVB pathway is inhibited in the absence of UBAP1. Tetherin levels in cells infected with a Vpu-defective HIV were high, similar to uninfected cells. Despite the restoration of tetherin levels in the UBAP1-depleted cells infected with wild-type HIV-1, viral release was not affected (Figure 2C). As expected, Vpu-defective virus release was approximately 100-fold less efficient than the wild-type virus in tetherin-expressing cells (Neil et al., 2008), and UBAP1 depletion did not affect this. These results show that, although Vpu induces targeting of tetherin for UBAP1-dependent ESCRT-mediated degradation, tetherin's ultimate destruction is not mandatory for Vpu to counteract its antiviral activity. Rather it suggests that Vpu interactions with tetherin (prior to UBAP1/ESCRT-I recruitment) inactivate the protein by committing it to an endosomal pathway from which it cannot escape back to the cell surface to inhibit virus release. Furthermore, the ability of tetherin to restrict HIV-1 delVpu release equivalently in UBAP1-depleted cells suggests that this knockdown has no significant effect on tetherin trafficking to the plasma membrane.

**Figure 3:**
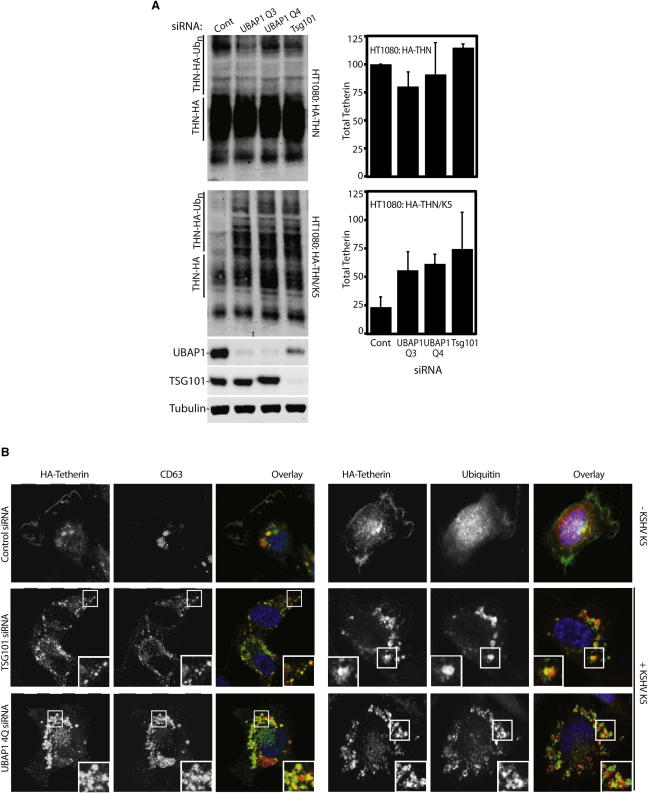
UBAP1 Depletion Prevents K5-Mediated Tetherin Degradation (A) Western blots of cell lysates from HT1080 cells stably expressing HA-tetherin alone (HT1080:HA-THN) or in combination with K5 (HT1080:HA-THN/K5) and treated with siRNAs against an irrelevant control (cont), TSG101 or UBAP1. THN-HA was detected with anti-HA antibody, with tubulin as a loading control and visualized using Li-Cor fluorescently coupled 650 and 800 nm secondary antibodies. The graphs show the percentage of mature tetherin levels, normalized to tubulin loading. Error bars indicate the standard deviation from the mean of three independent experiments. Lower panels show depletion of TSG101 and UBAP1, and tubulin as a loading control. (B) Confocal immunofluorescence showing localization of tetherin, CD63, and ubiquitin in cells treated with either control siRNA-, TSG101-, or UBAP1-specific siRNA. A higher magnification of the boxed areas is shown in TSG101- and UBAP1-treated panels. In the overlay panels, DNA is shown in blue, tetherin in green, and CD63 or ubiquitin in red. See also Table S3.

The second approach to determine UBAP1's function in endosomal sorting involved K5, a KSHV-encoded ubiquitin ligase that reduces cell surface expression of tetherin (Mansouri et al., 2009; Pardieu et al., 2010). We followed a knockdown approach in HT1080 cells stably expressing an HA-tetherin construct in addition to K5 (HT1080/HA-THN/K5). A quantitative western blot analysis was used to measure the total levels of HA-tetherin present in these cells. As expected, cells expressing K5 ubiquitin ligase had much lower levels of HA-tetherin (Figure 3A, lower panel). Transfection of siRNAs targeting UBAP1 prevented K5-mediated tetherin degradation, and tetherin protein levels were significantly rescued (lower panel in Figure 3A). Importantly, no changes in HA-tetherin were found when the same siRNAs were transfected into cells lacking K5. As a control, depletion of TSG101 also resulted in impairment of HA-tetherin degradation by K5 (Figure 3A). In agreement with the Vpu data, high-molecular-weight forms of tetherin, suggesting ubiquitinated species of the protein, were accumulated both in UBAP1 and TSG101 depleted cells (Figure 3A). Consistent with inhibition of endosomal sorting in UBAP1-depleted cells, HA-tetherin accumulated in intracellular punctae that display partial colocalization with the late endosomal marker CD63 and with ubiquitin (Figure 3B), a phenotype that is also observed in TSG101-depleted cells. Overall, these results show that UBAP1 is required for MVB sorting of an ubiquitinated cargo protein, namely tetherin.

### UBAP1 Is Not Required for HIV-1 Budding or Cytokinesis

**Figure 4:**
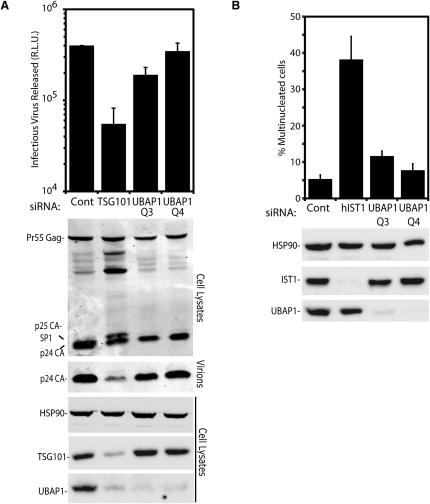
Role of UBAP1 in ESCRT-I Mediated HIV-1 Release and Cytokinesis (A) Infectious virus release upon coexpression of an HIV-1 provirus with an irrelevant siRNA (Cont), siRNA against TSG101 or two different siRNAs against UBAP1. Western blots show intracellular TSG101, UBAP1, and HSP90 (protein loading control) as well as intracellular (cell lysates) and virion-associated (virions) HIV Gag protein. (B) Quantification of cells with multiple nuclei after treatment with the irrelevant control siRNA (Cont), siRNA against hIST1 (positive control), or with either of the two different siRNAs against UBAP1. As for (A), western blots show siRNA-mediated silencing of the endogenous hIST1 and UBAP1 and HSP90 as a protein loading control. Error bars indicate the standard deviation from the mean of three independent experiments.

We also tested the role of UBAP1 in two other ESCRT-facilitated processes, namely HIV-1 budding and the resolution of the midbody during cytokinesis. Surprisingly, depletion of UBAP1 (more than 92% decrease in protein level) with two different siRNAs did not alter significantly HIV-1 budding, whereas TSG101 depletion inhibited infectious HIV-1 production as expected (Figure 4A). A similarly negative result was obtained in cytokinesis: although depletion of hIST1, an ESCRT-related protein essential for cytokinesis (Agromayor et al., 2009; Bajorek et al., 2009), resulted in cytokinesis failure and accumulation of multinucleated cells, UBAP1 depletion did not (Figure 4B). The lack of an effect of UBAP1 depletion on cytokinesis is consistent with results recently reported (Stefani et al., 2011). One possible explanation for this lack of phenotype is that the residual amount of UBAP1 protein in siRNA treated cells might be sufficient to support ESCRT-I function but the fact that similar levels of depletion in control experiments result in endosomal defects (shown above) argues against this possibility.

### UBAP1 Is a Ubiquitin-Binding ESCRT-I Subunit

**Figure 5:**
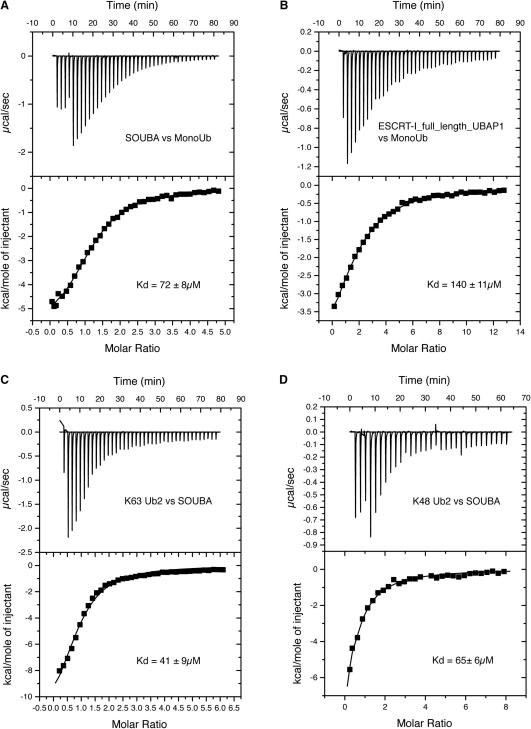
Ubiquitin Binding to UBAP1 Binding of human UBAP1 SOUBA domain (389–502) or UBAP1-containing ESCRT-I complex consisting of full-length UBAP1, VPS28 (full-length), TSG101 (198–390, i.e., lacking the UEV domain) and VPS37A (229–397, i.e., lacking the UEV domain). The experimental data (squares) were fit using a binding model for a single set of independent sites. The mean Kd and standard deviation were calculated from three independent measurements. (A) ITC titration of monoubiquitin into a cell containing the UBAP1 SOUBA domain (389–502), with the thermogram shown on top and integrated peaks shown on bottom. (B) ITC titration of monoubiquitin into a cell containing ESCRT-I with full-length UBAP1: thermogram (top) and integrated peaks (bottom). (C) ITC titration of the SOUBA domain into a cell containing K63 diubiquitin: thermogram (top) and integrated peaks (bottom). (D) ITC titration of the SOUBA domain into a cell containing K48 diubiquitin: thermogram (top) and integrated peaks (bottom).

The specific requirement of UBAP1 in endosomal sorting and the accumulation of ubiquitinated cargo after UBAP1 depletion from the cell suggested that UBAP1 might promote recognition of ubiquitinated cargo by ESCRT-I. We checked ubiquitin binding in solution in a label-free setup, using isothermal titration calorimetry (ITC). Using a recombinant fragment of UBAP1 consisting of the proposed UBA-containing region alone (residues 389 to 502), the Kd for the interaction with ubiquitin was about 70 μM (Figure 5A). This is on the higher-affinity end of the 20–1300 μM range commonly observed for UBA domains binding to monoubiquitin. Furthermore, this is a relatively high affinity among UBDs in the ESCRT pathway. We also measured binding of monoubiquitin to full-length UBAP1 in an ESCRT-I complex containing a TSG101 subunit with the N-terminal ubiquitin-binding UEV domain deleted in order to avoid ubiquitin binding by TSG101. The apparent Kd for the interaction between ESCRT-I/UBAP1 and monoubiquitin is about 140 μM (Figure 5B). Because many UBAs bind K48-linked diubiquitin with significantly higher affinity than monoubiquitin (typical Kds for K48-linked diubiquitin are 1–10 μM) and some UBAs bind K63-linked diubiquitin, we performed ITC experiments for label-free K48-linked or K63-linked diubiquitin binding to the SOUBA. We find that these diubiquitins bind the isolated SOUBA with an affinity comparable to that observed with monoubiquitin (Figure 5).

### Structural Analysis of the C-Terminal UBA-Containing Region of UBAP1

**Figure 6:**
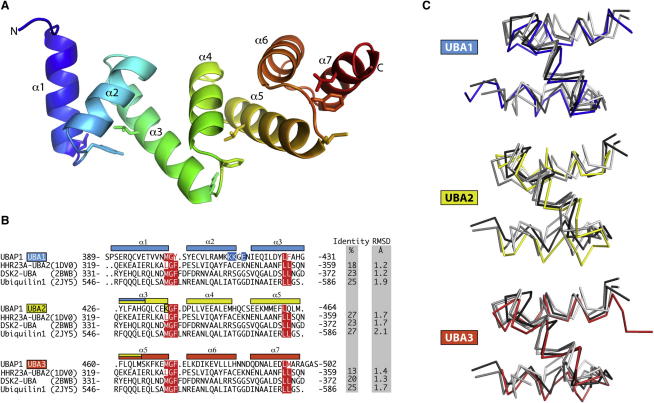
Overlapping UBAs Build a Compact SOUBA Domain (A) Overview of UBAP1 SOUBA domain (389–502), showing the tandem arrangement of the three overlapping UBA domains rainbow colored from N terminus (blue) to C terminus (red). (B) Sequence alignment of each of the three UBA domains of UBAP1 with three other well-characterized UBA domains. UBA signature motif residues are shown in red and represented as sticks in (A). Residues mutated in the SOUBA crystal structure are shown with a blue background. (C) Structural alignment of each of the three UBAP1 UBA domains with the three previously reported UBA domains present in the sequence alignment (B), namely Ubiquilin1 (2JY5) in light gray, DSK2 UBA (2BWB) in medium gray, and HHR23A UBA2 (1DV0) in dark gray. See also Table 1.

UBAP1 has two copies of an M/L-G-F/Y motif, which have recently been shown by mutagenesis to be important for endosomal sorting (Stefani et al., 2011). To gain insight into the organization and function of this region, we have determined its crystal structure and characterized its binding to ubiquitin in solution by NMR. Two constructs covering the UBAP1 C terminus, residues 381–502 and 389–502, produced ample protein but did not crystallize. Therefore, we used the surface entropy reduction approach (Derewenda, 2011) guided by the SERp Server (Goldschmidt et al., 2007) (http://services.mbi.ucla.edu/SER) to generate several mutants in which clusters of charged surface residues were mutated to alanine. One of these mutants, with 415-KKGE-418 mutated to AAGA, readily crystallized. The 1.65 Å resolution crystal structure of the UBA-containing C-terminal region from UBAP1 has two molecules in the asymmetric unit that are linked to each other through a disulfide bond. However, multiangle laser light scattering indicates that the molecule is a monomer in solution (data not shown). It appears that the disulfide-linked dimer is formed as the protein oxidizes during the crystallization process. The structure shows a single domain consisting of seven α helices arranged as a right-handed solenoid that contains three overlapping UBAs (UBA1, UBA2, and UBA3) (Figure 6A). We will refer to this, to our knowledge, novel domain as a solenoid of overlapping UBAs (SOUBA). The relationship of the SOUBA fold to conventional UBAs can best be described by regarding the third helix of one UBAs being also the first helix of the next UBA (Figures 6A and 6B). There are several examples of proteins that have multiple UBAs connected by linkers of low complexity that are probably structurally disordered. However, this is the first example of a rigid array of overlapping UBAs. The solenoid arrangement of the three UBAs forms a convex surface containing helices one and three from each UBA. The opposite face forms a concave surface built from the second helix from each UBA. The angles between helices in each of the UBAs are within the range observed for other UBAs. Consequently, each of the component UBAs align well with UBA structures from other proteins (Figure 6C).

### Interaction of the SOUBA with Monoubiquitin

**Figure 7:**
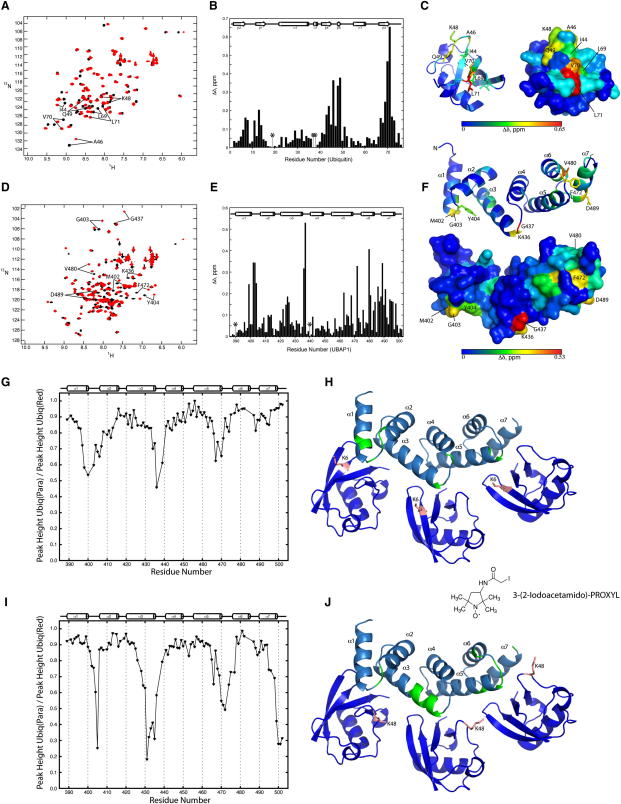
UBAP1 Interactions with Monoubiquitin Detected by NMR Chemical Shift Perturbations (A) Overlay of HSQC spectra of ^15^N-labeled monoubiquitin in the absence (black) and presence (red) of 2.5 molar equivalents of unlabeled UBAP1 SOUBA. Amide resonances with significant chemical shift changes have been annotated. (B) Plot of chemical shift perturbations (CSPs) for ^15^N-labeled monoubiquitin as a function of residue number. Proline residues (for which no data are available) have been assigned a value of 0 ppm and are marked by an asterisk. (C) CSPs for monoubiquitin mapped onto its structure. (D) Overlay of HSQC spectra of ^15^N-labeled SOUBA in the absence (black) and presence (red) of three molar equivalents of unlabeled monoubiquitin. (E) Plot of CSPs for ^15^N-labeled SOUBA as a function of residue number. (F) CSPs for SOUBA mapped onto its structure. (G–J) Paramagnetic relaxation enhancement effects in the SOUBA/Ub complex induced by the spin label attached to the K6C (G) and K48C (I) mutant ubiquitins. The graphs plot experimental PREs observed in SOUBA upon addition of the SL-ubiquitin against residue number. Significant paramagnetic effects are illustrated in green on a model of SOUBA bound to three molecules of ubiquitin for K6C-SL (H) and K48C-SL (J). The side chains of K6 and K48 (mutated to a Cys for spin labeling) are shown as salmon sticks.

Chemical shift perturbations (CSPs) of ^15^N-labeled ubiquitin on the addition of unlabeled SOUBA show shifts of residues in a hydrophobic patch on the surface of the ubiquitin that includes Ile44 and Val70 (Figures 7A–7C). Extensive structural analysis of diverse UBAs interacting with monoubiquitin has shown that the interaction surface on UBAs commonly includes two hydrophobic UBA patches, at the end of helix α1/loop 1 and on helix α3 (Zhang et al., 2008). Binding to K48-linked polyubiquitin typically includes an additional patch on α2 and the other side of α3. CSPs of the ^15^N-labeled SOUBA domain on addition of three molar equivalents of unlabeled monoubiquitin indicate that each of the three overlapping UBAs is influenced by ubiquitin binding (Figures 7D–7F). The residues showing the greatest perturbation lie along the convex face of the SOUBA domain and cover an extended hydrophobic surface (Figure 7F). The signature M/L-G-F/Y motifs are located along one side of the SOUBA domain, and CSPs suggest that these residues are involved in binding ubiquitin. This is consistent with the ubiquitin binding that has been reported for UBAs from other proteins (Long et al., 2008; Ohno et al., 2005; Varadan et al., 2005; Zhang et al., 2008). The second UBA in the UBAP1 SOUBA lacks the signature motif, and instead, it has a KGF sequence in the analogous position. Nevertheless, CSP mapping suggests that this KGF motif is also involved in ubiquitin binding, because it has the highest CSP of all residues upon ubiquitin binding. In addition to residues in these signature motifs, other residues along the same face of the SOUBA also show CSPs that are greater than 0.2 ppm. For UBA1 and UBA2, almost all of these residues are at the end of the first helix and on one face of the third helix within the UBAs. Although the “di-leucine” motif in the third helix of UBA domains is a second general signature motif, the equivalent residues in SOUBA (residues 427-LF in UBA1, 461-LQ in UBA2, and 495-LM in UBA3) do not show large CSPs on ubiquitin binding. The leucine in each of these motifs is involved in helical packing in the hydrophobic core. The largest CSPs that we observe in UBA1 and UBA2 are consistent with interactions that are typical of previously characterized UBA/monoubiquitin-interacting surfaces. However, the SOUBA UBA3 has a broader distribution of residues with large CSPs on all three of its helices, including a region that has been referred to as the “backside” of the third UBA helix. This could be due to conformational changes in the third UBA on ubiquitin binding, or due to an additional ubiquitin-binding site.

Paramagnetic relaxation enhancement (PRE) measurements were performed to probe directly the binding of each of the three UBA domains in SOUBA to ubiquitin and to investigate the relative orientation of SOUBA and monoubiquitin in the complex. A paramagnetic spin label (SL) was attached to a cysteine residue replacing a lysine in ubiquitin, in order to induce a strong signal attenuation in nuclei that are near the SL. Figure 7 depicts signal attenuations in the SOUBA in a complex with ubiquitin spin-labeled at either residue 6 (K6C mutant, Figures 7G and 7H) or residue 48 (K48C mutant, Figures 7I and 7J), compared to the same complexes in which the SL probe is inactivated by ascorbic acid. The results clearly show three regions of significant attenuation for K6C-SL ubiquitin (corresponding to the end of helix 1 and the loop between helix 1 and helix 2; the end of helix 3 and the loop between helix 3 and helix 4; the end of helix 5 and the loop between helix 5 and helix 6). Four regions of attenuation were observed on SOUBA with the K48C-SL ubiquitin (the loop between helix 1 and helix 2; the end of helix 3 and the loop between helix 3 and helix 4; the end of helix 5 and the loop between helix 5 and helix 6; and the end of helix 7). The additional signal attenuation observed for K48C-SL would suggest that the spin-label in K48C ubiquitin is close in space to two regions of each UBA motif. No paramagnetic effects were observed with D39C and S57C spin-labeled monoubiquitins. A model of the SOUBA bound to three monoubiquitin molecules reveals how the SL could achieve the effects observed (Figures 7H and 7J). The PRE measurements appear to match those observed by Zhang et al. (2008) when SL were used to investigate binding of the UBA domain of ubiquilin-1 with monoubiquitin. It is worth noting that the attachment of the SL to the mutant ubiquitins did not affect their ability to interact with SOUBA. Comparison of the NMR spectra of ^15^N-SOUBA in the presence of WT-Ub and SL-Ub (K6C, K39C, K48C, and K57C) revealed that the same CSP and relaxation regimes were observed.

## Discussion

The results presented in this study demonstrate that recombinant UBAP1 assembles with TSG101, VPS28, and VPS37 into a complex with a 1:1:1:1 stoichiometry and, importantly, this interaction is also observed in vivo with endogenous UBAP1 and physiological levels of the remaining ESCRT-I subunits. Codepletion of TSG101 and UBAP1 in cells treated with siRNA against TSG101 suggests that endogenous UBAP1 is stabilized by its interaction with TSG101. Moreover, UBAP1's ability to functionally recruit the ESCRT machinery in a TSG101-dependent manner provides further evidence for a functional link between UBAP1 and ESCRT-I. On the basis of these observations, we propose that UBAP1 is a stable subunit of human ESCRT-I. While this manuscript was in preparation, Woodman and colleagues have reported findings that are entirely consistent with our results (Stefani et al., 2011).

In a recombinant system, UBAP1 can form heterotetrameric complexes with TSG101, VPS28, and each of the four VPS37 subunits. Our results also show that transiently transfected VPS37A and VPS37B form UBAP1-containing complexes in 293T cells. Similarly, a recent study of UBAP1 expressed in HeLa cells showed complex formation with VPS37A. However, the same study did not detect formation of UBAP1 complexes with VPS37C (Stefani et al., 2011). This could mean that additional factors such as posttranslational modifications could modulate the association between UBAP1 and VPS37 subunits in cells. Little is known about the tissue distribution and cellular abundance of different VPS37 subunits or competing MVB12A and MVB12B, all of which could affect association with UBAP1.

UBAP1's function is exclusively required for degradation of ubiquitinated endosomal cargo but not for other ESCRT-I-mediated processes such as midbody abscission or viral budding. More specifically, we show that UBAP1-containing ESCRT-I complexes are parasitized by viral immunomodulatory proteins that target host membrane proteins for endosomal degradation, in particular the targeted destruction of the antiviral protein tetherin by both HIV-1 Vpu and KSHV K5 ubiquitin ligase. The essential role of UBAP1 in K5 function is entirely consistent with previous findings showing the ESCRT-dependent destruction of tetherin via a single lysine in its cytoplasmic tail (Mansouri et al., 2009; Pardieu et al., 2010). In contrast, the pathway involved in tetherin degradation and antagonism by Vpu has been less clear (Martin-Serrano and Neil, 2011). Although early studies suggested that Vpu induced proteasomal degradation of tetherin (Goffinet et al., 2009), more recent reports show that, in HIV-1-infected cells, tetherin is targeted to lysosomes (Douglas et al., 2009; Iwabu et al., 2009; Mitchell et al., 2009) and its inactivation is dependent on an association with Hrs (ESCRT-0) (Janvier et al., 2011). Our demonstration that UBAP1 is essential for Vpu-induced tetherin destruction shows that tetherin is degraded in the MVB pathway rather than by the proteasome. However, it is also clear that tetherin degradation is not a prerequisite for Vpu activity. Even though tetherin is not degraded in UBAP1-depleted cells and accumulates in endosomes, Vpu is still able to efficiently counteract tetherin's antiviral activity.

### A Model of Ubiquitin Binding to the SOUBA

At a mechanistic level, we demonstrate the ability of UBAP1 to promote the interaction of ESCRT-I with ubiquitin. A UBAP1-containing ESCRT-I complex is able to bind ubiquitin via the SOUBA domain located in the C-terminal region of UBAP1. The importance of this interaction is suggested by a reported frameshift mutation that precisely removes the SOUBA domain (S391Afs21X) in one case of familial frontotemporal lobar degeneration (Rollinson et al., 2009). Accordingly, mutations in the SOUBA domain disrupt sorting of endosomal cargo (Stefani et al., 2011). To our knowledge, the structure of this region shows a novel ubiquitin-binding fold, the SOUBA domain. The ability of UBAP1 to bind monoubiquitin may be important for the function of the ESCRT-I pathway, because half of conjugated ubiquitin is in the form of monoubiquitin or endcaps of polyubiquitin chains (Ziv et al., 2011), and monoubiquitination is a sufficient signal for vacuolar/lysosomal traffic (Stringer and Piper, 2011).

**Figure 8:**
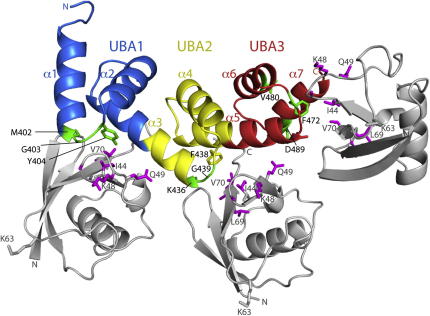
Model of Monoubiquitin Binding to the UBAP1 SOUBA The UBA1, UBA2, and UBA3 are colored blue, yellow, and red, respectively. UBAP1 residues showing a CSP >0.2 ppm upon binding to monoubiquitin are shown as green sticks. Three monoubiquitins have been docked on the basis of superposition of each UBA domain with the ubiquilin UBA domain in complex with ubiquitin (PDB ID 2JY6). Ubiquitin residues with CSPs >0.2 ppm upon binding to the UBAP1 SOUBA are shown as magenta sticks. The PRE and CSP measurements show that all three UBAs bind ubiquitin. The gradual addition of substoichiometric amounts of ubiquitin causes chemical shifts for residues in all three UBA motifs, suggesting that there is no preference for the binding of ubiquitin to one site versus another. The NMR data indicated that binding occurs at each of the sites, but cannot distinguish whether one, two, or three molecules are bound at the same time. Nevertheless, there appear to be no steric clashes in the model that would prevent all three ubiquitins binding simultaneously.

By superimposing the SOUBA domain on different UBAs whose structures have been solved in complexes with monoubiquitin, it is possible to generate a model of the SOUBA bound to ubiquitin. In this model, SOUBA can easily accommodate three monoubiquitins adjacent to each other, on the convex face of the domain (Figure 8). Each of the three UBAs engages with the hydrophobic patch of ubiquitin that includes Ile44 and Val70. Both the CSPs observed for ^15^N-labeled ubiquitin upon addition of an excess of unlabelled SOUBA (Figure 7C) and the relaxation induced by a paramagnetic probe attached to ubiquitin in complex with the SOUBA (Figures 7G–7J), are consistent with this model of ubiquitin binding. In this model, the orientations of the ubiquitins would not appear to allow the ubiquitins occupying adjacent sites on the SOUBA to be linked to each other either through K48 or K63 isopeptide bonds. This is consistent with our ITC results suggesting that these linkages bind no better than monoubiquitin to the SOUBA (Figure 5). Previous NMR studies of UBAs from several different proteins in the presence of K48-linked diubiquitin suggest a second binding site for the second ubiquitin involving a separate interface on the UBA (Sims et al., 2009; Song et al., 2009; Trempe et al., 2005; Varadan et al., 2005). The solenoid arrangement of the SOUBA would appear to block this type of binding, at least for the first two UBAs.

The NMR data demonstrate that each of the three UBAs is involved in ubiquitin binding, but it remains to be shown whether all three UBAs can bind ubiquitin simultaneously. We tried investigating the affinity of each UBA for ubiquitin by generating double point mutants in the (M/K)G(F/Y) motifs: we constructed three mutant SOUBAs, each having only one functional UBA. However, NMR HSQC spectra revealed that each of these mutants was unstructured, preventing further study. ITC results for the full-length UBAP1 in the ESCRT-I complex show a lower affinity for monoubiquitin than the isolated SOUBA domain. This might suggest a more restricted access to the SOUBA in the ESCRT-I complex. Because of the difficulties associated with producing sufficient quantities of diubiquitin, it was not possible to determine the affinity of UBAP1-containing ESCRT-I for K63-linked and K48-linked diubiquitin by ITC.

Because the shape of the SOUBA domain and the interaction sites mapped by NMR lead to a model in which three bound ubiquitins are aligned and close enough to be attached via some other type of linkage than K48 or K63, it will be exciting to examine UBAP1 affinities for other polyubiquitin linkages. Alternatively, in addition to providing a grip on monoubiquitinated cargo, the SOUBA domain could enable avid interaction with multiply monoubiquitinated cargoes, or monoubiquitinated clustered cargoes. All of these are frequent passengers in the ESCRT-dependent sorting to MVBs.

## Experimental Procedures

### Plasmid Construction

Human *UBAP1* was amplified by PCR from IMAGE clone 40011243. All deletions and point mutant derivatives were generated by PCR in a similar way. All of the constructs used in the study are listed in Table S1.

### Expression and Purification of Recombinant ESCRT-I Proteins

Genes coding for human VPS28 (full length or 1-122), TSG101 (198-390), VPS37A (229-397), and UBAP1 (full length or deletion variants with a C-terminal His6-tag) were cloned in a polycistronic coexpression vector as described elsewhere (Teo et al., 2006). The constructs expressed are listed in Table S1. The expression and purification was performed as described in Supplemental Experimental Procedures.

### Expression and Purification of SOUBA

Human SOUBA domain (UBAP1 389-502) either wild-type or containing the K415A, K416A, and E418A triple mutation was cloned in the pOPTH(tev) expression vector downstream of an N-terminal His_6_-tag followed by a TEV protease cleavage site. The expression and purification were performed as described in Supplemental Experimental Procedures.

### Crystallization, Data Collection, and Refinement of SOUBA

Seleno-methionine crystals of the human SOUBA domain (UBAP1 389-502) with the K415A, K416A, and E418A triple mutation designed to reduce surface enthropy charge (using SERp server: http://services.mbi.ucla.edu/SER/) were used for crystallization (see Supplemental Experimental Procedures and Table 1).

### Isothermal Titration Calorimetry

Assays were performed using a Microcal ITC200, in 20 mM Tris-HCl (pH 7.5), 100 mM NaCl, and 1 mM TCEP. The ITC cell contained either the SOUBA domain (UBAP1(389-502)) at 150 μM or full-length UBAP1 associated with VPS28 (full-length), TSG101 (198-390, i.e., lacking the UEV domain), and VPS37A (229-397, i.e., lacking the UEV domain), at 75 μM and was titrated with 38 injections of monoubiquitin (SIGMA U6253) at 3 mM. Assays on K63-linked and K48-linked diubiquitins were performed having diubiquitin in the cell at 100 μM, titrated by 38 injections of the SOUBA domain at 2.3 mM. Data were fit with a binding model employing a single set of independent sites.

Expression and purification of paramagnetically labeled monoubiquitin was performed as described in Supplemental Experimental Procedures.

### NMR Spectroscopy

Wild-type SOUBA (UBAP1_389-502) samples prepared for NMR spectroscopy experiments were typically 1.0 mM in 90% H2O and 10% D2O in PBS with 10 mM DTT. All spectra were acquired with either a Bruker Advance 700 or a DRX600 spectrometer at 20°C, and referenced relative to external sodium 2,2-dimethyl-2-silapentane-5-sulfonate (DSS) for proton and carbon signals, or liquid ammonium for nitrogen. Assignments were obtained using standard NMR methods with ^13^C/^15^N-labeled and ^15^N-labeled samples. Backbone assignments were obtained using the following standard set of 2D and 3D heteronuclear spectra: ^1^H-^15^N HSQC, HNCACB, CBCA(CO)NH, HACACO, HNCO, CCCONH, and ^1^H-^13^C HSQC. HSQC titrations were performed using 0.5 mM ^15^N-labeled UBAP1 and varying concentrations of unlabeled ubiquitin. Similar titrations were performed with 0.3 mM ^15^N-labeled ubiquitin and unlabeled UBAP1. Amide CSPs were calculated as follows:

$$\Delta \text{δ}\;={\left[{\left(\Delta {\text{δ}}_{\text{H}}\right)}^{2}+{\left(\frac{\Delta {\text{δ}}_{\text{N}}}{5}\right)}^{2}\right]}^{1/2},$$

where Δδ_H_ and Δδ_N_ are the observed chemical shift changes for ^1^H and ^15^N, respectively. Paramagnetic experiments were performed using sample containing 0.3 mM SOUBA and 0.3 mM chemically modified monoubiquitin.

### Generation of Stable Cell Lines

HT1080/THN-HA K5 cells have been described elsewhere (Pardieu et al., 2010). To generate HeLa OSHA-TSG101, 293T cells were transfected with 100 ng of pHIT-VSVG, 700 ng of MLV-GagPol, and 200 ng of the pCMS28 retroviral packaging vector for 48 hr. Viral-containing supernatants were collected and used to transduce HeLa. Selection with puromycin (200 ng/ml) was applied 48 hr later, and cells were passaged under continual selection.

### Coprecipitation Assays

These were performed as described in Supplemental Experimental Procedures.

### HIV Infectivity Assays

Cells (293T) were transfected with 250 ng of the YFP fusions and 300 ng of pNL/HXB using polyethylenimine. Culture supernatants, collected 48 hr after transfection, were clarified by low-speed centrifugation, and particles present in 250 μl were obtained by centrifugation through a 20% sucrose cushion at 14,000 rpm for 2 hr. Viral protein content in cell and particle lysates was analyzed by western blotting with an anti-Gag antibody. Alternatively, indicator HeLa-TZM-bl cells (CD4^+^, CXCR4^+^, CCR5^+^, HIV-1 LTR- LacZ) (Derdeyn et al., 2000) were infected with 1 μl of supernatant and 48 hr later, β-galactosidase activities in cell lysates were measured using the chemiluminescent detection reagent Galacto-Star (Applied Biosystems).

To assay inhibition of viral production by siRNA-mediated depletion of cellular UBAP1, 293T cells were initially transfected with 50 pmol of siRNA using Dharmafect1 (Dharmacon) and were split the next day. Forty-eight hours after initial transfection, cells were cotransfected with 50 pmol of siRNA and the HIV proviral plasmid using lipofectamine 2000 (Invitrogen). The detailed sequence of the siRNA oligos used is provided in Table S2. The plasmids and method for the trans-complementation assay have been described previously (Martin-Serrano et al., 2001).

### Multinucleation Assays

The multinucleation assays for HeLa cells were performed as described in Supplemental Experimental Procedures.

### Vpu-Mediated Tetherin Degradation Assay

Subconfluent 293T cells stably expressing tetherin were seeded in a six-well plate and 2 hr after plating were transfected with 50 pmol of siRNA using Dharmafect-1 (Dharmacon). Forty-eight hours later, cells were reseeded and transfected again with 50 pmol of siRNA. Six hours after the second RNAi transfection, cells were infected with HIV-1 wild-type or Vpu-deficient at an MOI of 2. At 48 hr after infection, the cells were lysed on ice for 30 min in buffer containing 50 mM Tris-HCl (pH 7.4), 150 mM NaCl, complete protease inhibitors (Roche), and 1% digitonin (Calbiochem). After removal of the nuclei, the resulting supernatants were incubated with 1 μg/ml mouse anti-tetherin (eBiosciences) for 2 hr at 4°C before addition of 40 μl of protein G-agarose (Invitrogen) for a further 3 hr. The beads were then washed four times in lysis buffer containing 0.1% digitonin. After the final wash, beads were resuspended in water and treated with the protein deglycosylation kit from New England Biolabs under denaturing conditions as specified by the supplier. Samples were resuspended in SDS-PAGE loading buffer and cell lysates and immunoprecipitates were then western blotted for tetherin using rabbit anti-BST2 (kindly provided by K. Strebel through the NIH ARRP).

### K5-Mediated Tetherin Degradation Assay

Total tetherin-HA levels were analyzed by western blot of cell lysates after siRNA treatment of HT1080 cells stably expressing HA-tetherin alone (HT1080:HA-THN) or in combination with K5 (HT1080:HA-THN/K5). Tetherin-HA was detected with anti-HA antibody, with tubulin as a loading control, and visualized using Li-Cor fluorescently coupled 650 and 800 nm secondary antibodies.

### Western Blot Analysis

Cell extracts, as well as virion lysates, were separated on 10% or 12% polyacrylamide gels and transferred to nitrocellulose membranes. A list of antibodies used is provided in Table S3.

### Immunofluorescence Microscopy

HT1080:HA-THN and HT1080:HA-THN/K5 cells were treated with siRNA as described above for the multinucleation assays. At 24 hr after transfection, the cells were fixed in 4% paraformaldehyde, permeabilized in 0.1% Triton X-100, and immunostained using a rabbit anti-HA antibody (Rockland) and mouse anti-CD63 (Developmental studies Hybridoma Bank. University of Iowa) or mouse anti-Mono and polyubiquitylated conjugates (clone FK2) (Enzo Life Sciences) followed by the appropriate donkey secondary antibodies coupled to Alexa 488 and 594 fluorophores (Invitrogen). Nuclei were visualized using Hoechst 33258 and coverslips were mounted in Mowiol. Images were taken using a Leica AOBS SP2 confocal microscope.

## Figures and Tables

**Table 1:** Crystallographic Statistics

	Se-Met Crystal For Structure Refinement
**Data collection**	

Space group	P1
Cell dimensions	
*a*, *b*, *c* (Å)	34.40, 43.48, 59.41
α, β, γ (°)	102.57, 96.47, 113.24
Wavelength	0.98
Resolution (Å)	38.3 (1.65)^a^
*R*_sym_ or *R*_merge_	0.055 (0.54)
*I* / σ*I*	9.9 (1.9)
Completeness (%)	95.9 (94.4)
Redundancy	3.9 (3.9)

**Refinement**	
Resolution (Å)	1.65
No. reflections	36253
*R*_work_ / *R*_free_	0.1494/0.1907
No. atoms	2101
Protein	1822
Ligand/ion	37
Water	242
*B*-factors	34
Protein	32
Ligand/ion	66
Water	47
R.m.s deviations	
Bond lengths (Å)	0.0165
Bond angles (°)	1.536

a The values in parentheses are for the highest resolution shell, 1.74 Å to 1.65 Å.

## Supplemental Information

Document S1. Figure S1, Tables S1–S3, and Supplemental Experimental Procedures
