# Supplementary material for: The UBAP1 Subunit of ESCRT-I Interacts with Ubiquitin via a SOUBA Domain
Source: Structure. 2012 Mar 7;20-135(3-3):414–28. doi: 10.1016/j.str.2011.12.013 (PMC3314968; doi:10.1016/j.str.2011.12.013)
Supplement: Document S1. Figure S1, Tables S1–S3, and Supplemental Experimental Procedures [file mmc1.pdf]

Structure, Volume *20*

Supplemental Information

The UBAP1 Subunit of ESCRT-I Interacts with Ubiquitin via a SOUBA Domain

Monica Agromayor, Nicolas Soler, Anna Caballe, Tonya Kueck, Stefan M. Freund, Mark D. Allen, Mark Bycroft, Olga Perisic, Yu Ye, Bethan McDonald, Hartmut Scheel, Kay Hofmann, Stuart J.D. Neil, Juan Martin-Serrano, and Roger L. Williams

Inventory of Supplemental Information

Supplemental Figures

Figure S1, UBAP1 is a component of ESCRT-I, related to Figure 1

Supplemental Tables

Table S1, Plasmid constructs used in this study, related to Figure 1

Table S2, siRNA sequences used in this study, related to Figure 2.

Table S3, Primary antibodies used in this study, related to Figure 3.

Supplemental Experimental Procedures

Supplemental Figures

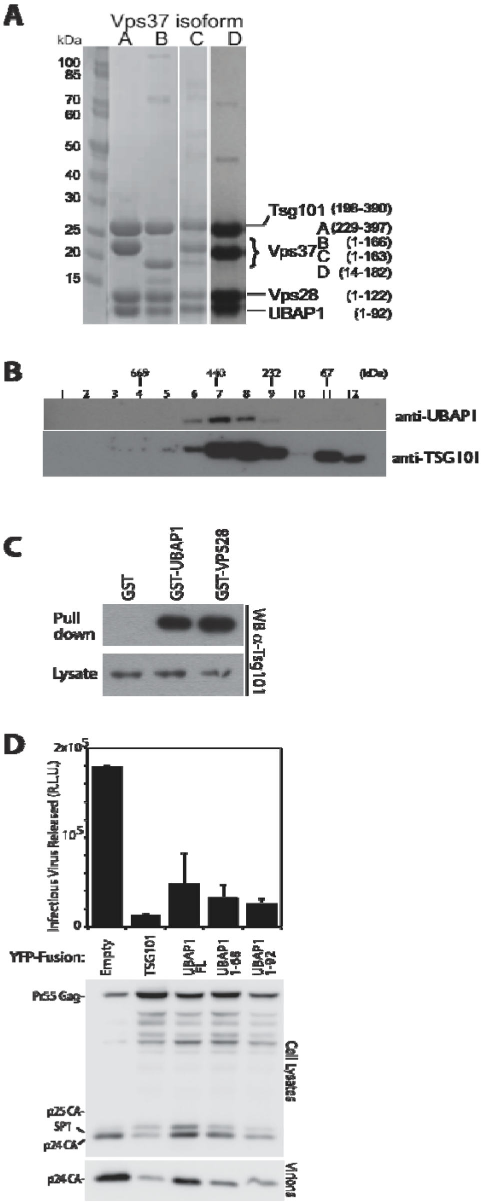

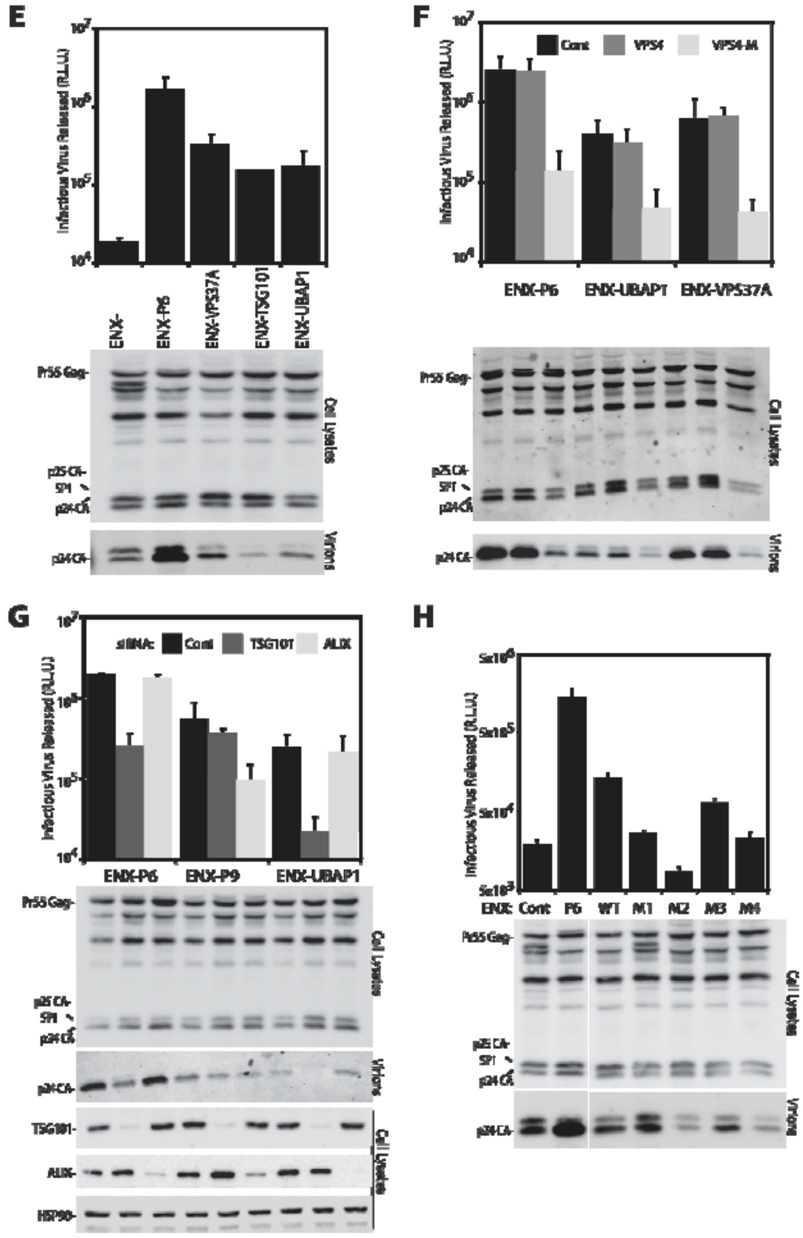

Figure S1, related to Figure 1. UBAP1 is a component of ESCRT-I

(A) The cores of all VPS37 isoforms can form stable ESCRT-I complexes with UBAP1 when co-expressed in *E. coli* and co-purified by affinity chromatography and gel filtration. Coomassie stained SDS-PAGE of the purified complexes are shown.

(B) Detection of endogenous UBAP1 and TSG101 in HEK293 cell lysate fractionated by gel filtration chromatography. Fractions were analysed by SDS-PAGE and

immunoblotted with either anti-UBAP1 (top) or anti-TSG101 antibody (bottom). Positions of the molecular weight size markers are indicated above fraction numbers. (**C**) Co-precipitation assays showing binding of endogenous TSG101 to over-expressed UBAP1 and VPS28 fused to GST. 1% of the starting cell lysate and 10% of the volume eluted from the beads (pull-down) were analyzed by western blot with anti-TSG101 antibody.

(**D**) Effects of over-expression of YFP-tagged UBAP1 constructs on HIV-1 release. 293T cells were transfected with HIV-1 proviral DNA and plasmids expressing YFP fused to UBAP1 full-length (UBAP1-FL) or truncated (UBAP1 1-68 and UBAP1 1-92). YFP control (empty) and YFP-TSG101 were included as negative and positive controls respectively. Infectious virus release was measured after infection of TZM-bl cells (*R.L.U.* relative luminescence units). Error bars indicate the standard deviation from the mean of three independent experiments. Cell lysates and extracellular virions were analyzed by western blot with anti-HIV Gag antibody.

(**E-H)** Trans-complementation experiments showing that UBAP1 functionally recruits ESCRT-I. While not demonstrating a crucial role of UBAP1 in ESCRT-I-mediated viral budding, these experiments show that, when artificially recruited, UBAP1 can act as an adaptor that bridges Gag to the ESCRT machinery and in particular to ESCRT-I. (E) Infectivity assays following co-transfection of cells with a L-domain mutant HIV-1 proviral plasmid and plasmids expressing an HIV-1 Gag protein in which p6 was either deleted (ENX-), intact (ENX-P6), or replaced with VPS37A (ENX-VPS37A), TSG101 (ENX-TSG101), or UBAP1 (ENX-UBAP1). Infectious virus release was measured after infection of TZM-bl cells, and it is expressed as relative luminescence units (R.L.U.). Error bars indicate the standard deviation from the mean of three independent experiments. Cell lysates and extracellular virions generated were analyzed by western blot with anti-HIV Gag antibody. The positions on the blot of the precursor Pr55Gag, cleavage intermediate p25 (CA-SP1) and mature p24 (CA) proteins are indicated. (F-G) Overexpression of a catalytically

inactive form of the ATPase VPS4, whose activity is essential for all ESCRT-mediated functions, strongly inhibited UBAP1-mediated complementation The same trans-complementation assay was done in the presence of either Myc tagged VPS4, HA-VPS4 mutant or empty vector (control) expressing plasmids, as indicated (F) or RNAi specific for TSG101, ALIX or non-coding control (G). The bottom images in (G) show the siRNA-mediated silencing of the endogenous TSG101 and ALIX and a protein loading control as determined by Western blot analysis of the cell lysates with anti-TSG101, anti-ALIX, and anti-HSP90. (H) A budding deficient HIV-1 provirus and plasmids expressing HIV-1 Gag protein fused to UBAP1 wildtype (ENX-WT) or bearing point mutations (ENX-M1 to M4) were co-transfected in 293T cells. Infectious HIV-1 virion production was measured by inoculation of TZM-bl indicator cells and is expressed as relative luminescence units (R.L.U.). Error bars indicate the standard deviation from the mean of three independent experiments. Cell lysates and extracellular virions generated were analyzed by western blot with anti-HIV Gag antibody. As negative and positive controls plasmids encoding a Gag protein in which p6 was either deleted (ENX-Cont) or intact (ENX-P6) were used respectively.

Supplemental Tables

Table S1: Plasmid constructs used in this study, related to Figure 1.

Construct abbreviation	Subunit composition
ESCRT_UBAP-FL	HsVPS28 full length, HsTSG101_198-390, HsVPS37A_229-397, HsUBAP1 full length with a C-terminal His6-tag in pOPC vector (Plasmid pOP571)
ESCRT_UBAP-1-92	HsVPS28_1-122, HsTSG101_198-390, HsVPS37A 229-397, HsUBAP1_1-92 with C-terminal His6-tag in pOPC vector (Plasmid pOP589)
UBAP1_381-502	HsUBAP1_381-502 with an N-terminal His6 tag in pOPTH(tev) vector (Plasmid pOP673)
UBAP1_389-502	HsUBAP1_389-502 with an N-terminal His6 tag in pOPTH(tev) vector (Plasmid pOP674)
UBAP1_(389-502)/K415A/ K416A/E418A	HsUBAP1_389-502 with an N-terminal His6 tag in pOPTH(tev) vector (Plasmid pNS11)
GST-UBAP1	HsUBAP1 full length in pCAG-GST vector
GST-VPS28	HsVPS28 full length in pCAG-GST vector
GST-TSG101	HsTSG101 full length in pCAG-GST vector
HA-UBAP1	HsUBAP1 full length in pCR3.1/HA vector
HA-UBAP1 M1	HsUBAP1 full length with mutation LDD/AAA in residues 17-19, in pCR3.1/HA vector
HA-UBAP1 M2	HsUBAP1 full length with mutation VPF/AAA in residues 20-22, in pCR3.1/HA vector
HA-UBAP1 M3	HsUBAP1 full length with mutation F28A, in pCR3.1/HA vector
HA-UBAP1 M4	HsUBAP1 full length with mutation E59A, in pCR3.1/HA vector
HA-VPS37A	HsVPS37A full length in pCR3.1/HA vector
HA-VPS37B	HsVPS37B full length in pCR3.1/HA vector
Myc-VPS28	HsVPS28 full length in pCR3.1/Myc vector
Myc-VPS4	HsVPS4A full length in pCR3.1/Myc vector
Myc-VPS4 DN	HsVPS4A full length with K173Q point mutation in pCR3.1/Myc vector

Table S2: siRNA sequences used in this study, related to Figure 2.

Protein	Sense sequence	Source
ALIX	GAAGGAUGCUUUCGAUAAAUU	Dharmacon
hIST1	TCGCCTTAAACTATTGGAGAA	Qiagen
TSG101	CCUCCAGUCUUCUCUCGUC	Dharmacon
UBAP1-Q3	CTCGACTATCTCTTTGCACAT	Qiagen
UBAP1-Q4	CAGCTAAAGTTGGTCTACCTA	Qiagen
Cont	ON-TARGET plus siControl non-targeting pool (D-001810-10-20)	Dharmacon

Table S3: Primary antibodies used in this study, related to Figure 3.

Antibody	Source
Mouuse anti-Tubulin (clone DM1a)	Sigma
Mouse anti-HIV-1 p24, clone 183-H12-5C	National AIDS Research and Reference Reagent Program
Mouse anti-TSG101, clone 4A10	Abcam
Mouse anti-CD63	Developmental studies Hybridoma Bank. University of Iowa
Mouse anti human HLA ABC:FITC, clone W6/32	AbD Serotec
Mouse anti-human CD317 (tetherin)	eBioscience
Mouse anti-Mono and polyubiquitiylated conjugates (clone FK2)	Enzo Life Sciences
Mouse anti-HA	Covance
Rabbit anti-Bst2 (tetherin)	Gift from K. Strebel (Laboratory of Molecular Microbiology, NIH)
Rabbit anti-HA	Rockland Immunochemicals
Rabbit anti-UBAP1	ProteinTech Group
Rabbit anti-VPS37A	ProteinTech Group
Rabbit anti-hIST1	ProteinTech Group
Rabbit anti-HSP90	Santa Cruz

Supplemental Experimental Procedures

Expression and purification of recombinant ESCRT-I proteins - Expression was done in C41(DE3)RIPL *E. coli*. Cells (6 L) were grown at 37 °C in 2XTY medium containing 100 µg/mL ampicillin to an optical density of 0.8 at 600 nm. Expression was induced with 1 mM IPTG. After induction, cells were incubated over-night at 12 °C, then pelleted by centrifugation at 4000 g. The pellets were resuspended in 160 ml of buffer W1 (20 mM Tris pH8.0 (4 °C), 50 mM potassium phosphate pH 8.0 (4 °C), 100 mM NaCl, 15 mM imidazole, 0.05% Triton X-100, 1 mM TCEP). Cells were lysed by pulsed sonication for 10 minutes. The lysate was ultracentrifuged at 95000 g for 45 min and passed through a 0.2 µm filter before loading on a HisPrep™ FF 16/10 Column (GE Healthcare) equilibrated in buffer W1. The column was washed with buffer W1, followed by the buffer W2 (same as W1 but without Triton X-100) and eluted with 400 mM imidazole in buffer W2. Fractions containing the four ESCRT-I subunits were pooled and loaded on a 5 mL Hitrap heparin HP column coupled to two tandem 5 ml Hitrap Q columns (GE Healthcare) equilibrated with buffer A (20 mM Tris pH 6.8 (20 °C), 1 mM DTT). The complex is not retained on the heparin column but binds the Q-resin. The coupled columns were washed with 1 volume of buffer A before the heparin column was removed. Protein was eluted from the Q column using a 12 column volume gradient of 0-100% buffer B (20 mM Tris pH6.8 (20 °C), 1 M NaCl, 1 mM DTT). Fractions were analyzed on SDS-PAGE, and those containing a 1:1:1:1 stoichiometric ratio of the 4 subunits were pooled and concentrated to a volume of 2 mL using a 10 kDa MWCO Amicon Ultra-15 concentrator (Millipore). Concentrated proteins were loaded on a HiLoad 16/60 Superdex 200 column (GE Healthcare) equilibrated in 20 mM Tris-HCl pH 7.5, 100 mM NaCl, 2 mM DTT. Fractions corresponding to a tetrameric ESCRT-I complex containing UBAP1 were pooled and concentrated to 12.9 mg/ml.

Expression and purification of SOUBA - Expression was done as described above, except that the cells were induced at OD_{600nm} of 0.9 for 4 hours at 37 °C. Following purification on a Ni-resin, fractions containing the SOUBA domain were pooled and subjected to TEV cleavage at mass ratio (TEV/protein) of 1/30 overnight at 4 °C in order to remove the N-terminal 6His-tag. The cleaved product was passed through a HisTrap™ FF 5 mL to separate it from the 6His-labelled TEV protease and then loaded on a 5 ml Hitrap Q columns (GE Healthcare) equilibrated with buffer A (20 mM Tris pH 8, 1 mM DTT). Protein was eluted from the Q column, using a 10 column volume gradient of 0-100% buffer B (20 mM Tris pH 8, 1 M NaCl, 1 mM DTT). Fractions containing the protein were concentrated and loaded on a HiLoad 16/60 Superdex 75 column (GE Healthcare) equilibrated in 20 mM Tris-HCl pH 7.5, 100 mM NaCl, 2 mM DTT. SDS-PAGE of the fractions showed a pure 13.5 kDa band corresponding to the SOUBA domain, which was pooled and concentrated to 21 mg/ml.

Seleno-methionine substituted protein was grown in methionine-requiring B834(DE3) cells. Pre-cultures grown in 2XTY medium containing 100 µg/mL ampicillin were used to inoculate M9 minimal medium supplemented with 0.4 % w/v glucose, 100 µg/mL ampicillin, 2 mM MgSO4, 25µg/mL w/v FeSO4, 1µg/mL Riboflavin, 1µg/mL Niacinamide, 0.1µg/mL pyridoxine monohydrochloride, 1µg/mL_thiamine), 0.04 mg/ml of each amino acid except methionine and 0.04 mg/ml seleno-L-methionine. After ~10 hrs at 37°C the cultures were induced with 1 mM IPTG and incubated for an additional 4 hours at 37 °C before harvesting. Subsequent purification proceeded as for unlabeled protein.

Crystallization of the SOUBA domain - Seleno-methionine crystals of the human SOUBA domain (UBAP1 389-502) with the K415A, K416A and E418A triple mutation designed to reduce surface enthropy charge (using SERp server: http://services.mbi.ucla.edu/SER/) were grown by mixing 0.2 µL of the concentrated protein with 0.2 µL of a solution containing 645 mM Na/K tartrate, 271 mM LiSO4,

100 mM CHES pH 9.5, 2 mM TCEP, 5% glycerol, and 0.1 µL of seeds from native crystals obtained in similar conditions. The crystals grew in space group P1 with unit cell dimensions given in Table 1. Data collection and refinement were carried out as described in Supplementary experimental procedures.

Crystallographic data collection and refinement - The diffraction data were collected at ESRF BM30 using a selenomethionine-substituted crystal and SAD methodology (Table 1). Sites for 7 selenomethionines were located using HySS and refined with SOLVE (Zwart et al., 2008). An initial model for the domain was generated using RESOLVE. This initial model was manually adjusted with COOT (Emsley and Cowtan, 2004) and refinement was carried out using PHENIX. Data collection and refinement statistics are given in Table 1. There are two molecules in the asymmetric unit that are related by a non-crystallographic two-fold axis. The two molecules in the asymmetric unit are linked to each other through a disulfide bond between two-fold related copies of residue Cys452. The structure shows density for two oxidized selenomethionines.

Purification of diubiquitins – K48-linked and K63-linked diubiquitins were prepared as described previously (Dong et al., 2011).

Expression and purification of paramagnetically labelled monoubiquitin – Wild-type monoubiquitin cloned into a modified pRSETa (Invitrogen) vector was mutated using a Quickchange mutagenesis kit to generate the following clones: K6C, D39C, K48C and S57C. The proteins were expressed in C41 cells and purified using ammonium sulphate precipitation, acid denaturation and gel filtration, and were kept in a reduced state throughout by using buffers containing 10 mM DTT. The mutant ubiquitins were passed though a NAP10 column to remove the DTT and immediately labeled by incubation with a 100-fold molar excess of 3-(2-iodoacetamido)-PROXYL (Sigma-Aldrich). The reaction was allowed to proceed for 12 h at room temperature before the excess label was removed using a NAP10 column. To obtain spectra

of chemically modified but not paramagnetic samples, spin-labeled samples were reduced by the addition of 10 mM ascorbic acid. All paramagnetic and chemically modified but non-paramagnetic ubiquitin samples were dialysed into PBS buffer prior to use.

Coprecipitation Assays - 293T cells were transfected with GST, HA and myc expression vectors (1 μg of each) using polyethylenimine (Polysciences, Inc). 36 hr later, cells were harvested and lysed in 1 ml of 50 mM Tris.HCl, pH 7.4; 150 mM NaCl; 5 mM EDTA; 5% glycerol; 1% Triton X-100 and a protease inhibitor cocktail (complete mini-EDTA free, Roche). Clarified lysates were incubated with glutathione-sepharose beads (GE Healthcare) for 3 hr at 4 ºC and washed three times with wash buffer (50 mM Tris.HCl, pH 7.4; 150 mM NaCl; 5 mM EDTA; 5% glycerol; 0.1% Triton X-100). The bead-bound proteins were eluted by boiling in 100 µl of sodium dodecyl sulfate sample buffer and analyzed by western blotting with anti-HA monoclonal antibody. The same approach was followed for coprecipitation of proteins fused to One-strep tag with the difference that clarified lysates from cells stably expressing the tagged proteins were incubated with Strep-Tactin superflow plus (Qiagen).

Multinucleation Assays – HeLa cells (25000) were seeded in a 48 well plate and 2 hr after plating were transfected with 50 pmol of siRNA targeting either Luciferase, UBAP1 or hIST1 using Dharmafect-1 (Dharmacon). 48 hr later cells were reseeded onto glass coverslips and transfected again with 50 pmol of siRNA for another 48 hr. After RNAi treatment, cells were fixed with 3% paraformaldehyde for 15 min, permeabilised with PBS/0.1% Triton X-100 for 5 min and then stained with monoclonal anti-Tubulin in PBS/1% BSA for 2 hr. Alexa594 conjugated secondary antibody was applied in PBS for 1 hr. Nuclei were visualized using Hoechst 33258 and coverslips were mounted in Mowiol. Images were taken using a Leica AOBS SP2 confocal microscope. 300 cells per coverslip were scored for the presence of

more than one nucleus. Cells unambiguously connected by midbodies were considered multinucleated.

Supplementary references

Dong, K. C. et al. (2011). Preparation of distinct ubiquitin chain reagents of high purity and yield. Structure *19*, 1053-1063.

Emsley, P., and Cowtan, K. (2004). Coot: model-building tools for molecular graphics. Acta Crystallogr D Biol Crystallogr *60*, 2126-2132.

Zwart, P. H. et al. (2008). Automated structure solution with the PHENIX suite. Methods Mol Biol *426*, 419-435.
